# Fungicidal activities of soil humic/fulvic acids as related to their chemical structures in greenhouse vegetable fields with cultivation chronosequence

**DOI:** 10.1038/srep32858

**Published:** 2016-09-06

**Authors:** Meng Wu, Mengya Song, Ming Liu, Chunyu Jiang, Zhongpei Li

**Affiliations:** 1 State Key Laboratory of Soil and Sustainable Agriculture, Institute of Soil Science, Chinese Academy of Sciences, Nanjing 210008, China; 2 Graduate University of Chinese Academy of Sciences, Beijing 100049, China

## Abstract

In the background of rapid expansion of plastic greenhouse vegetable production in China, many environmental risks have emerged in recent years. In this study, the soils with a chronosequence in greenhouse vegetable fields were collected and the soil humic acids (HAs) and fluvic acids (FAs) were extracted and purified. The soil HAs and FAs were found to show inhibition activities against phytopathogenic fungi for the first time. Fourier transform infrared spectroscopy was performed to investigate the chemical structures of HAs and FAs. The variation of relative peak areas indicated the chemical structure of HAs become more complex and stable under continuous cultivation. The PCA analysis showed HAs and FAs could be distinctly separated from each other and cultivation years mainly determined the variation. Mantel test and RDA analysis indicated the active components (aliphatic peaks for HAs and COOH, OH peaks for FAs) had positive correlation with the inhibition rates of HAs and FAs against phytopathogenic fungi. According to our research, the active fungicidal components in soil HAs and FAs decreased along with the extension of cultivation years, which made the soil suffer more risk to phytopathogenic fugi. So we believe continuous cultivation too many years in PGVP systems is inadvisable.

Globally, the use of greenhouses in agriculture is expanding to 3.7 million hectares over the past two decades and plastic greenhouses occupied ~95% of the area covered by vegetable greenhouses^1^. As a country with the largest population, vegetable cultivation under plastic greenhouse conditions is expanding rapidly in China over the last 20 years in the background of rapid urbanization^2^. Many farmers have shifted from conventional grain cropping to plastic greenhouse vegetable production (PGVP) systems. PGVP has played an important role in increasing vegetable production and rural incomes^3^,^4^. However, the unique PGVP practices such as excessive chemical fertilizer, high temperatures, and high cropping indexes can cause serious environmental problems. For example, accumulations of heavy metals and producing more greenhouse gases in PGVP systems have become common problems^1^. High application of fertilizer has resulted in obvious change of soil chemical properties including soil salinization and acidification^5^,^6^, decreased soil enzyme activities and nitrification capacity^7^, and change in microbial community^8^. Continuous cropping under plastic greenhouses could also lead to the degeneration of soil productivity and crop yields^9^. Converting cereal land to greenhouse vegetable field also affects the soil organic matter (SOM) content^10^,^11^. But the structure change of organic matter in plastic greenhouses vegetable field is still not clear.

Humic acid (HA) and fulvic acid (FA) are important components of organic matter in soil, interacting with plant growth and microorganism activity. For FAs, people mainly focus on their antioxidant activities^12^, while HAs exhibited many biological activities which were found to be useful in plant cultivation, pharmacology and ecology. HAs have a well-acknowledged capability to induce plant development, especially for root systems^13^,^14^,^15^. Besides HA itself displaying biological activity, some active substrate such as *bacillus thuringiensis* could bound to HAs^16^, and then the HAs could show insecticidal activity. Ansorg and Rochus found HAs extracted from different soils and two synthetic HAs showed antimicrobial activity against many human pathogenic bacteria such as *Str. Pyogenes and Ps. Aeruginosa*^17^. While Yarkova found peat and coal HAs showed insignificant inhibition of the growth of bacteria (*St. aureus*, *S. Enteritidis* etc.)^18^, but a chemical modification of the initial structure of HAs by acylation could increase the biological activity of native peat and lignite HAs. That indicated the origin of HAs considerably influenced their antimicrobial activities and the main reason may be related to the difference in the chemical structure of the HAs. Muscolo *et al.* also indicated that different activities of humic substances were related to their diverse chemical composition^19^. So studying the structure difference of HAs will help us to understand the variation of their biological activities. The Fourier transform infrared (FT-IR) spectroscopy is widely used to study the soil and HAs chemical structures^20^,^21^.

Although HAs displayed many biological activities, there are no reports about the fungicide activity of humic substances against plant pathogenic fungi. As we know, soil-borne diseases are caused by phytopathogenic microbes (fugi accounted for the majority) in the soil, which may damage plants upon penetration of the root or basal stem^22^. If there are antifungal substrates in soil, soil-borne diseases will be less to happen. In this paper, the humic acid and fulvic acids extracted from greenhouse vegetable soils with different cultivation years were evaluated for their fungicidal activities against phytopathogenic fungi. The temporal variation of chemical structures of humic acids and fulvic acids and their relationship with fungicidal activities in plastic greenhouses vegetable field was also investigated.

## Results

### Characteristics of soil organic matter

From the results shown in Supplementary Table S1, it is indicated that converting rice/wheat field to greenhouse vegetable land substantially affected the soil chemical properties. Soil pH was decreased 1.5–2.3 after cultivating 6–20 years and the soil was changed to be quite acidic from neutral soil (CK). The SOC and TN were significantly increased after converting cropland to PGVP for 3–10 years (*P* < 0.05) and then decreased from 10 years to 20 years. The SOC and TN firstly increased for some years because of large fertilizer inputs. However, continuous cultivation made soil degenerate and that may cause SOC and TN decrease. Similar result was found in Northeast China, the content of organic matter in 0–60 cm soil first increased (in 0–15 years) and then decreased (in 15–25 years) along with vegetable planted years^11^. So the soil type and cultivation years may both affect the accumulation of organic matter in PGVP systems. The humic to fulvic acid ratio C_HA_/C_FA_ was the frequently used quality criterion of SOM^23^. As shown in Supplementary Table S1, the value of the ratio C_HA_/C_FA_ significantly increased for 3 and 6-years cultivated greenhouse vegetable soils as compared to CK (*P* < 0.05). However, the C_HA_/C_FA_ ratio decreased from 1.22 in the 3-year cultivated soil to 0.89 in the 20-year cultivated soil and the C_HA_/C_FA_ ratio of 10 and 20-year cultivated soil showed no significant difference with CK (*P* > 0.05).

### FT-IR spectroscopy analysis of HAs and FAs

**Figure 1:**
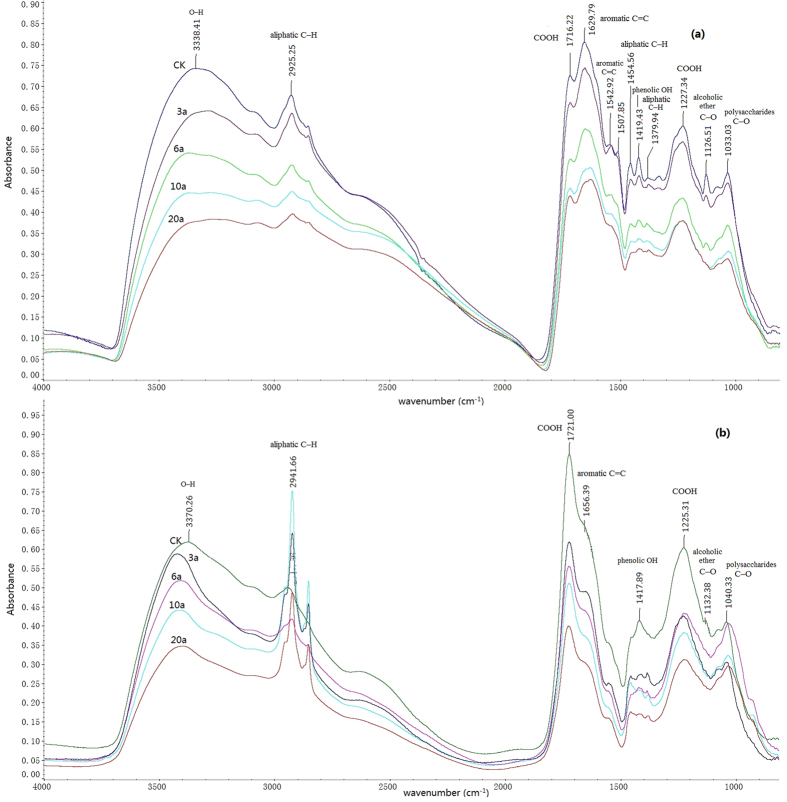
The FT-IR spectra of soil humic acids and fulvic acids. Representative FT-IR spectra are shown for humic acids (**a**) and fulvic acids (**b**) (n = 3).

The FT-IR spectra of HAs or FAs from different cultivation years showed a similar spectroscopic pattern. The dominant functional groups in the FT-IR spectra obtained from HAs and FAs are illustrated in Fig. 1. The peaks of the FT-IR spectra were assigned according to the previous work^24^,^25^. The following peaks occurred in the spectra obtained from both HAs and FAs: The broad band between 3400 and 3300 cm^−1^ belongs to H-bonded OH groups. The peaks occurring at about 2940–2920 and 2850 cm^−1^ are attributed to the asymmetric and symmetric aliphatic C–H stretching of CH_2_ groups. An intense peak at 1725–1710 cm^−1^ is attributed to C=O stretching of COOH. The peak at 1450–1456 cm^−1^ is caused by aliphatic C–H deformation. A band at 1420–1411 cm^−1^ is assigned to O–H deformation and C–O stretching of phenolic OH. A peak between 1384–1376 cm^−1^ can be attributed to C–H deformation of CH_2_ and CH_3_ groups and/or to antisymmetric stretching of COO− groups. The band at 1227–1224 cm^−1^ is assigned to C–O stretching and O–H deformation of COOH groups and C–O stretching of aryl ethers and phenols. A band at about 1040–1030 cm^−1^ is attributed to C–O stretching of polysaccharides and polysaccharide-like substances, and/or Si–O of silicate impurities. The peak of Si–O stretching vibration at 1080 cm^−1^ is not appearing in our samples, so we can confirm that silicate impurities are quite scarce in our samples and the peak at 1040–1030 cm^−1^ can be assigned to polysaccharides.

There are also some peaks occurring in HAs can’t be found in FAs. Bands occurring at 1628–1650 cm^−1^ are attributed to aromatic C=C skeletal vibrations and C=O stretching of quinine. A peak at 1545–1540 cm^−1^ belongs to the aromatic C=C stretching. A peak at 1130–1126 cm^−1^ is attributed to C–O stretching of various alcoholic and ether groups. It’s worth noting that the peak at 1130–1126 cm^−1^ occurs only in HA-CK, HA-3a and HA-6a while disappears in HA-10a and HA-20a. The obvious differences of FT-IR spectra between HAs and FAs indicate that HAs contain more complex aromatic structures than FAs.

Here, to quantitatively study the structural differences of HAs and FAs with different cultivation years in greenhouse vegetable field, the relative peak area (area of one peak divided by the sum of area of all investigated peaks) of dominant peaks on the FT-IR spectra were calculated and described in Table 1. The results indicated that the relative peak areas at 2930 cm^−1^ (3010–2800 cm^−1^) which represent the aliphatic C–H and 1720 cm^−1^ (COOH) in HAs were smaller than that of FAs. In contrast, the relative peak areas of 1630 and 1540 cm^−1^ (aromatic C=C) in HAs were bigger than that of FAs. The relative peak areas of 2930, 1450 cm^−1^ (aliphatic peaks) in HAs decreased along with cultivation years. While relative peak areas of 1630 cm^−1^ (aromatic peak) in HAs increased from CK to 3-years greenhouse vegetable soil and also increased along with cropping years. The relative peak areas of 1720 cm^−1^ in FAs decreased from CK to 3-years cultivated soil and also decreased along with cultivation years. The index I_ar_ was assigned as a ratio between the integrated peak areas of the aromatic C=C absorption bands (1630 cm^−1^) and the areas of aliphatic C–H bands (2930 cm^−1^) and was found to increase with the prolongation of cultivation years.

**Figure 2:**
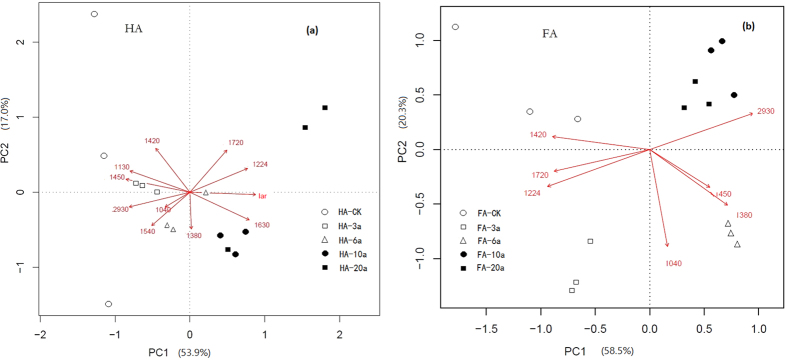
A biplot of principal component analysis of FT-IR integrated peak areas of HAs and FAs with different cultivation years.

Principal component analysis (PCA) was applied on the FT-IR data to investigate qualitative differences within the HAs and FAs. The first principle component (PC1) and the second principle (PC2) of the PCA explain 53.9 and 17.0%, respectively, of variance in 11 spectroscopic indices of HAs (Fig. 2a). While axes 1 and 2 of the PCA explain 58.5 and 20.3% of variance in 7 spectroscopic indices of FAs (Fig. 2b). From Fig. 2a, it could be found that HA-CK, HA-3a with negative scores on PC1 are separated from HA-10a, HA-20a with positive scores on PC1. The peak areas at 2930, 1450 and 1130 cm^−1^ have strong negative weightings and the peak area of 1224, 1630 cm^−1^ and I_ar_ have strong positive weighting on axis 1, which all obviously contribute to the separation. Similarly, as shown in Fig. 2b, the peak areas at 1224, 1420 and 1720 cm^−1^ have strong negative weightings and the peak area of 2390 cm^−1^ have strong positive weighting on PC1, which separates FA-CK and FA-3a from the other treatments with more cultivation years in greenhouse vegetable fields. FA-CK is also separated from FA-3a in opposite quadrants with positive scores on PC2. As shown by the distribution of different FT-IR peaks in the PCA plot, all sample are different from each other and are distributed along with PC1 following with different cultivation years, which means the cultivation years mainly determine the difference of the FT-IR peaks of HAs and FAs.

### The fungicidal activity of HAs and FAs

**Figure 3:**
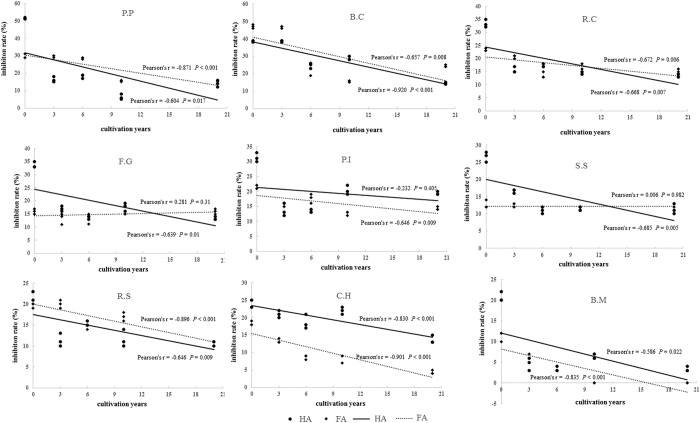
Linear regressions between cultivation years in greenhouse vegetable fields and the inhibition rate of HAs and FAs against diffierent phytopathogenic fungi. Two-tailed Pearson’s correlation analysis was also used to investigate the relationships between the cultivation years and the inhibition rates (n = 3). 0 year refer to samples HA-CK and FA-CK (never cultivated in greenhouse).

The HAs and FAs are naturally resistant to many phytopathogenic fungi in different degree according to the biological results (Table 2). Especially, the HA from conventional rice/wheat field (HA-CK) exhibited more than 50% inhibition rate against *Physalospora piricola* and more than 30% inhibition rate against *Botrytis cinerea*, *Rhizoctonia cerealis*, *Fusarium graminearum* and *Phytophthora infestans*. While the FA-CK exhibited much lower inhibition rate than HA-CK against the tested phytopathogenic fungi except *Botrytis cinerea*. It can be found that the HAs with different cultivation years in greenhouse vegetable fields all exhibited significant lower inhibition rate than HA-CK (*P* < 0.05) except HA-3a displayed the same activity as HA-CK against *Botrytis cinerea*. The HA-20a showed the lowest fungicidal activity against most of the tested phytopathogenic fungi. The linear correlation plots (Fig. 3) with Pearson’s correlation analysis indicated that the inhibition rates of HAs decreased significantly along with the prolongation of cultivation years (*P* < 0.05) against most of tested fungi except *Phytophthora infestans*. The inhibition rates of FAs showed the same negative correlation with cultivation years (*P* < 0.05) against most of tested fungi except *Fusarium graminearum* and *Sclerotinia sclerotiorum*.

### The correlations of fungicide activity and chemical structure of HAs and FAs

**Figure 4:**
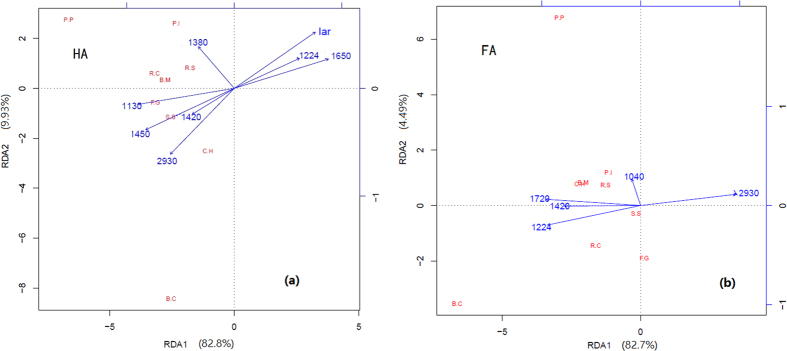
Redundancy analysis of inhibition rates against different phytopathogenic fungi and FT-IR peak areas of HAs and FAs.

To assess the relationships between the fungicide activity and FT-IR peak areas of HAs and FAs, Mantel test and redundancy analysis (RDA) were applied. Mantel tests were performed to examine the relationships between the FT-IR peak areas and the total inhibition rate. The relations between the abundance of total chosen FT-IR peaks and total inhibition rates were both significant (*P* < 0.001) for HAs and FAs. For HAs, the peaks occurring at 2930, 1630, 1450, 1420, 1380, 1224, 1130 cm^−1^ and I_ar_ were significantly correlated with inhibition rate against phytopathogenic fungi (*P* < 0.05, Table 3). For FAs, the peaks at 2930, 1720, 1420, 1224 and 1040 cm^−1^ were significantly correlated with inhibition rate (*P* < 0.05, Table 3). The peaks significantly correlated with the inhibition rates were picked to further perform a redundancy analysis (RDA). The results of RDA showed that the peaks at 1130, 1450, 1630 cm^−1^ and I_ar_ of HAs and the peaks at 1224, 1720, and 2930 cm^−1^ of FAs have stronger effects on inhibition rate (Fig. 4), which were similar to those of Mantel tests. Axis 1 explained 82.8% and 82.7% of the variation in the inhibition rates of HAs and FAs. The inhibition rates of HAs and FAs against different phytopathogenic fungi all appeared at the negative axis RDA1. From the biplots, it is indicated the peaks at 1130, 1380, 1420, 1450, 2930 cm^−1^ have positive correlation and the peaks at 1224, 1630 cm^−1^, I_ar_ have negative correlation with most of the inhibition rates of HAs. The peaks at 1040, 1224, 1420, 1720 cm^−1^ have positive correlation and the peak occurring at 2930 cm^−1^ have negative correlation with the inhibition rates of FAs.

## Discussion

The C_HA_/C_FA_ ratio, as a humification parameter, reveals the degree of humification of SOM^26^. Continuous cultivation in greenhouse vegetable fields decreased the C_HA_/C_FA_ ratio, suggesting that cultivation caused preferential degradation of large-molecular humic substances and/or a neo-synthesis of small-molecular humic substances. Similar results were reported by Sun *et al.*^27^, who found a decrease of 33% in the C_HA_/C_FA_ ratio after 200 years cultivation for the black soil in the maize field. In the process of cultivation, agricultural practices such as increasing input of chemical fertilizer tend to exert a substantial impact on soil structure and the composition of SOM^28^. In the present study, C_HA_/C_FA_ significantly increased after converting cropland to greenhouse vegetable field for 3–6 years (*P* < 0.05) and it is indicated that short-term heavy fertilization in greenhouse vegetable field could increase the degree of humification of organic matter. The C_HA_/C_FA_ ratio decreased from 3 years to 20 years, indicating that soil humification decreased under prolonged cultivation.

As we know, the relative proportions of the integrated areas of FT-IR peaks have been widely used to study structural changes in HAs^29^,^30^. The variation of the relative peak areas could indicate the structural differences of soil HAs and FAs. The proportion of HAs aliphatic peaks (2930, 1450 cm^−1^) decreased and HAs aromatic peak (1630 cm^−1^) increased along with cultivation years demostrated the chemical structure of HAs become more complex with cultivation years. Demyan *et al.* indicated the ratio between the integrated peak areas of 1630 cm^−1^ and 2930 cm^−1^ (I_ar_) positively correlated with the ratio of stable C and to labile C and was used as an indicator of SOM stability^24^. Here, the increase of I_ar_ also indicated HAs become more stable along with cultivation years. While for the FAs, the active group (COOH) decreased along with cultivation years. The PCA plot of FT-IR peak areas showed that HAs and FAs could be separated at axis 1 following with different cultivation years, which indicated the temporal variation of soil HAs and FAs in PGVP systems.

Humic substances were found to exhibit many biological activities such as anti-inflammatory and pro-inflammatory properties^31^. K-humates isolated from oxihumolites could alleviate tobacco mosaic virus infection^32^ and humic acids from brown coals displayed biostimulation activity^33^. However, there are no reports about the fungicidal activity of humic subtances before. According to our research results, the soil HAs and FAs all have the ability to inhibit many phytopathogenic fungi. That means the soil has the nature to resist some soil-borne diseases. The inhibition rates of HA-CK were much higher than FA-CK against tested fungi except *Botrytis cinerea*, which indicated that more complicated compounds in HA displayed higher inhibition rates than the simple compounds in FA. It was detrimental to the fungicidal activity of HA when converting the conventional rice/wheat production to PGVP systems and the fungicidal activity of HAs also further decreased after perennial cropping in greenhouse vegetable fields. The inhibition rates of FAs showed similar but smaller variation than HAs among the cropland soil and soils with different cultivation years in greenhouse vegetable fields. The conversion of traditional croplands to PGVP systems could result in some potential harm on soil health. In the previous study, researchers mainly focused on accelerated phosphorus and heavy metals accumulation^10^, secondary salinization and acidification^5^,^6^, lower N use efficiency^34^, and a decrease in soil microbial diversity^7^. But no one has assessed the effect on their autologous fungicidal activity. According to our results, the decrease of fungicidal activity of HAs and FAs could reflect the soil degradation process in PGVP systems and is also a soil health risk. So we believe the fungicidal activity of HAs and FAs could be treated as an indicator of soil health condition.

The structures of HAs and FAs are quite different from each other and functional groups (aromatic, aliphatic, hydroxyl and carboxyl groups) have different influence on the fungicidal activities of HAs and FAs. Mantel test and RDA analysis were conducted to discern possible linkages between fungicide activity and the chosen FT-IR peak areas. The results showed that the typical aliphatic peaks (1130, 1450, 2930 cm^−1^) had positive correlation with the inhibition rates of HAs, while the aromatic peaks (1224, 1630 cm^−1^) and I_ar_ had negative correlation with the inhibition rates of HAs. The typical OH and COOH peaks (1224, 1420, 1720 cm^−1^) have positive correlation with the inhibition rates of FAs, while the relative stable CH_2_ group peak (2930 cm^−1^) have negative correlation with the inhibition rates of FAs. The HAs contain much stable aromatic constituents and the aliphatic peaks of FT-IR could be treated as the active components of HAs. As FAs contain little aromatic constituents, the typical OH and COOH peaks could be regarded as the active components of FAs. Our research demonstrated that the active components of HAs and FAs have positive correlation with their fungicidal activities against phytopathogenic fungi. The decrease of active components accounts for the reduction of fungicidal activities of HAs and FAs along with the extension of cultivation years in greenhouse vegetable fields.

It has been found that PGVP systems were likely to increase crop diseases, which may lead to yield reduction^35^. According to our research, the reduction of fungicidal components in soil HAs and FAs under continuous cultivation make the soil more susceptible to phytopathogenic fungi and that may account for crop diseases. Considering the long-term risk of soil health, continuous cultivation too many years in PGVP systems is inadvisable.

In summary, the soil HAs and FAs were found to have fungicidal activity against many phytopathogenic fungi for the first time. The fungicidal activity of HA decreased when converting the conventional cropland to greenhouse vegetable fields. The inhibition rates of HAs decreased regularly along with cultivation years and that reflected the temporal variation for fungicidal activity of HAs in PGVP systems. The inhibition rates of FAs showed the same negative linear correlation with cultivation years. C_HA_/C_FA_ increased after converting cropland to PGVP for 3 years and decreased from 3 years to 20 years, which suggested soil humification could increase in short-term but will decrease under prolonged cultivation in PGVP system. The relative areas of HAs aromatic peaks and the index I_ar_ increased along with cultivation years demonstrated the chemical structure of HAs become more complex and stable with cultivation years. While for the FAs, the COOH group decreased along with cultivation years. The PCA analysis showed that cultivation years mainly determined the difference of the FT-IR peaks, which indicated the temporal variation for the chemical structure of HAs and FAs. RDA analysis demonstrated the chemical structural changes determined the variation of fungicidal activity. The active aliphatic constituents of HAs and the active groups (OH and COOH) of FAs account for their fungicidal activities.

Compared with conventional cropland, PGVP is under conditions of yearly continuous production with high-intensity use of fertilizer, irrigation and energy. After a few years of protected cultivation in PGVP system, the chemical structure of HAs and FAs substantially changed and make the soil more susceptible to phytopathogenic fungi. Considering the significant change in fungicidal activities and relative FT-IR peak areas between 3-year and 6-year humic substances (*P* < 0.05), continuous cropping more than 3 years in PGVP systems is not recommended and some practices (such as fallowing or removing the plastic films for a year) need to be implemented to maintain the soil health and minimize the soil decline in plastic greenhouse vegetable fields.

## Materials and Methods

### Site description

The field site is located in Xinzhuang Town, Changsu City, Jiangsu Province, China (31°33′N, 120°38′E), where the climate is a subtropical monsoon with around 240 frost-free days. The annual mean temperature and accumulated precipitation is 15.5 °C and 1,038 mm, respectively. The site soil is classified as Anthrosols, a gleyed paddy soil derived from lacustrine deposits. In this area, greenhouse vegetable production has developed rapidly in place of the conventional rice/wheat rotations in recent years. For each planting season, the main fertilizer used were NPK compound fertilizer (22% N, 16% P_2_O_5_, 16% K_2_O) and together with slight urea with the mean annual application rates approximately equivalent to 825–1000 kg N, 600 kg P_2_O_5_ and 600 kg K_2_O ⁄ ha. While in local croplands, the mean annual application rates approximately equal to 400 kg N, 200 kg P_2_O_5_ and 200 kg K_2_O ⁄ ha. Spray irrigation is widely used in the plastic greenhouse vegetable fields.

### Soil sample

Soil samples from the plastic greenhouse vegetable fields managed by local farmers with 3, 6, 10 and 20 cropping years were collected for the study. All the selected greenhouses have the same tillage conditions and vegetable varieties. The soil from an adjacent conventional rice/wheat field was used as the control (CK). In each field, the soils were taken from three randomly chosen plastic covered greenhouses as triplicate samples. And in each greenhouse, the topsoil (0–15 cm depth) was collected at random and mixed to give a composite sample. The soil samples were air-dried and sieved (<2 mm) and plant debris and roots were removed as much as possible prior to further analysis.

Soil organic carbon (SOC) was determined by the Tyurin method^36^, total N (TN) by the semimicro Kjeldahl method^37^ and total P (TP) and K (TK) were digested by HF–HClO_4_ and determined by molybdenum-blue colorimetric and flame photometry, respectively. Soil available N (AN) by alkali hydrolysable method^38^, soil available P (AP) by Olsen method^39^, and soil available K (AK) by NH_4_OAC extraction^40^, pH by the potentiometric method.

### Preparation of humic acids and fulvic acids

Humic substances were extracted from 50 g soils to analyze the chemical structure and the fungicidal activities. Isolation and purification methods were carried out according to the method implemented by the International Humic Substances Society. A 50 g sample was extracted by 0.1 M NaOH with the liquid/solid ratio of 10 under N_2_, The solution was shaken for 6 h. The extracted humic substances were separated into HA and FA fractions by acidifying the extract to pH = 1 using 6 M HCl. The supernatant (FA) and the precipitated fraction (HA) were separated by centrifugation at 4000 rpm for 20 minutes. The HA was washed by 50 mL 0.1 M HCl two times and the supernatant was combined with FA fraction. The HA was re-dissolved by 0.1 M NaOH and diluted in a volumetric flask. A portion of HA and FA were measured for organic C concentration. The organic C concentration of the FA fraction was determined using a Jena 3100c TOC analyzer (Analytik Jena AG, Jena, Germany). The organic C concentration of the HA solutions was measured after diluting with 1/15 M KH_2_PO_4_ to obtain a pH about 6. The C_HA_/C_FA_ ratio was calculated according to the organic C content of HA and FA.

The HAs were washed by the repetition of re-precipitation with diluted HCl, centrifugation and re-dissolution in 0.1 M NaOH. Then the HA precipitates were shaken two times with 20-fold volume of 0.1 M HCl: 0.3 M HF (volume ratio 1:1) for 4 h at 25 °C to remove inorganic contaminants, and dialyzed against distilled water until Cl^−^ was no longer detected with AgNO_3_. The FAs were purified by an adsorption resin (XAD-8, Supelco Co.), and the alkaline elutions (0.1 M NaOH) were passed through H^+^-saturated cation exchange resin (AG-MP-50, Bio-rad Co.). The HA and FA powders were obtained by freeze-drying.

### Diffuse reflectance Fourier transform infrared spectroscopy

The FT-IR spectroscopy analysis of HAs and FAs was performed according to Demyan *et al.*^26^. Mid-infrared spectra were recorded on a Nicolet iS10 (Thermo Scientific, USA) Fourier transform spectrometer using a potassium bromide (KBr) beam splitter. The HAs and FAs were grounded into powders and sieved through a 2-μm sieve and dried at 32 °C. Then 2.0 mg of the samples were mixed with 200 mg KBr powder. The spectra were recorded in the mid-infrared range (4000–400 cm^−1^) by combining 32 individual scans at a resolution of 8 cm^−1^. Spectral pre-processing included atmospheric correction for carbon dioxide and water, baseline correction and vector normalization. The FT-IR spectra were recorded in absorbance units (AU). Peak area integration on the corrected spectra was performed using the spectral processing software Nicolet Omnic version 9 (Thermo Nicolet).

### Fungicidal Activity

The *in vitro* fungicidal activities of HAs and FAs against nine phytopathogenic fungi, *Physalospora piricola* (*P*.*P*), *Botrytis cinerea* (*B*.*C*), *Rhizoctonia cerealis* (*R*.*C*), *Fusarium graminearum* (*F*.*G*), *Phytophthora infestans* (*P*.*I*), *Sclerotinia sclerotiorum* (*S*.*S*), *Rhizoctonia solani* (*R*.*S*), *Cercospora arachidicola Hori* (*C*.*H*), *Bipolaris maydis* (*B*.*M*) were tested by means of mycelium growth assays according to previous report^41^. The HAs and FAs were dissolved in DMSO, and diluted to 50 mg/L with water containing emulsifier (200 ug/mL). Then, the test solution (1 mL) was poured into sterile culture plates (9 cm diameter), and agar culture medium (9 mL) was added. The solution without HAs or FAs (1 mL) and agar culture medium (9 mL) were used as controls. The inocula, 4 mm in diameter, were removed from the margins of actively growing colonies of mycelium, placed in the centers of the above plates, and incubated at 25 ± 1 °C. The diameter of the mycelium was measured for 72 h. Each treatment was performed in triplicate. The inhibition percent was used to describe the control efficiency of the compounds.

Inhibition percent (%) = (average hyphal diameter in the control − average hyphal diameter in the treatment)/average hyphal diameter in the control.

### Statistical analysis

The difference between the fungicide activity and chemical properties of each sample was evaluated by using one-way ANOVA with Duncan method, and *P* < 0.05 was considered to be significant using SPSS 20.0 software (IBM). Linear regression and two-tailed Pearson’s correlation analysis was used to investigate the relationships between the cultivation years and the inhibition rates of HAs and FAs. Principal components analysis (PCA) was performed on the standardized relative peak area data of HAs and FAs. Mantel test was employed to explore the relative FT-IR peak areas that significantly correlated to the inhibition rates against nine phytopathogenic fungi. These peak areas were used to establish an environmental variables matrix for redundancy analysis (RDA). Subsequently, RDA was conducted to investigate the relationships between inhibition rates and relative peak areas which were standardized to reduce the influence of descriptor scale on RDA results. The Mantel test, PCA and RDA analyses were conducted in the “R” language environment and “vegan” package (Development Core Team 2006).

## Additional Information

**How to cite this article**: Wu, M. *et al.* Fungicidal activities of soil humic/fulvic acids as related to their chemical structures in greenhouse vegetable fields with cultivation chronosequence. *Sci. Rep.* **6**, 32858; doi: 10.1038/srep32858 (2016).

## Supplementary Material

Supplementary Information

## Figures and Tables

**Table 1:** Relative proportions of the integrated areas of the peaks in FT-IR spectra.

Samples	2930 cm^−1^	1720 cm^−1^	1630 cm^−1^	1540 cm^−1^	1450 cm^−1^	1420 cm^−1^	1380 cm^−1^	1224 cm^−1^	1130 cm^−1^	1040 cm^−1^	I_ar_
HA-CK	19.7 ± 0.6 a	2.30 ± 0.38 ab	32.1 ± 1.3 c	0.85 ± 0.12 ab	3.75 ± 0.15 a	1.45 ± 0.98 a	1.00 ± 0.63 a	31.9 ± 1.0 c	1.82 ± 0.28 a	5.12 ± 0.15 a	1.63 ± 0.02 c
HA-3a	19.7 ± 0.1 a	1.69 ± 0.04 b	34.3 ± 0.2 b	1.13 ± 0.15 a	3.09 ± 0.14 b	1.26 ± 0.14 a	0.32 ± 0.02 b	32.9 ± 0.24 ab	0.82 ± 0.15 b	4.81 ± 0.09 a	1.75 ± 0.01 c
HA-6a	18.3 ± 0.3 ab	1.84 ± 0.06 b	36.2 ± 0.4 a	0.76 ± 0.12 ab	2.86 ± 0.14 b	1.02 ± 0.09 a	0.67 ± 0.06 ab	32.4 ± 0.69 a	0.86 ± 0.10 b	5.18 ± 0.71 a	1.98 ± 0.01 b
HA-10a	17.7 ± 0.8 bc	1.98 ± 0.27 b	37.3 ± 0.7 a	1.06 ± 0.26 a	2.17 ± 0.33 c	1.11 ± 0.25 a	0.88 ± 0.14 ab	33.8 ± 0.63 bc	—	4.81 ± 0.19 a	2.11 ± 0.06 bc
HA-20a	16.6 ± 1.4 c	3.07 ± 1.13 a	37.3 ± 0.3 a	0.42 ± 0.46 b	1.82 ± 0.02 d	0.86 ± 0.14 a	0.76 ± 0.04 ab	34.4 ± 0.87 c	—	4.80 ± 0.48 a	2.26 ± 0.18 a
FA-CK	21.5 ± 5.0 B	19.8 ± 1.2 A	—	—	0.56 ± 0.14 A	1.68 ± 0.72 A	0.34 ± 0.18 B	54.3 ± 6.0 A	—	1.56 ± 0.28 D	—
FA-3a	20.6 ± 2.5 B	16.0 ± 1.5 B	—	—	0.46 ± 0.04 A	0.89 ± 0.08 B	0.42 ± 0.04 AB	56.8 ± 1.8 A	—	4.92 ± 0.23 A	—
FA-6a	43.5 ± 3.1 A	12.6 ± 0.3 C	—	—	2.15 ± 0.04 C	0.61 ± 0.04 B	0.58 ± 0.01 A	39.0 ± 0.5 BC	—	3.68 ± 0.42 B	—
FA-10a	49.7 ± 3.1 A	10.2 ± 0.2 D	—	—	0.69 ± 0.06 B	0.39 ± 0.01 B	0.44 ± 0.08 AB	34.0 ± 0.7 C	—	2.56 ± 0.06 C	—
FA-20a	44.8 ± 2.4 A	10.9 ± 0.3 D	—	—	0.73 ± 0.04 B	0.48 ± 0.03 B	0.44 ± 0.03 AB	39.9 ± 2.2 B	—	2.84 ± 0.13 C	—

Mean values and the standard deviation are given. Small letters indicate significant differences between the humic acids, and capital letters indicate significant differences between the fulvic acids. The significant differences are calculated by ANOVA with Duncan test (*P* < 0.05, n = 3), labeled from highest to lowest value. “—” means no such peaks exist in the sample. Index I_ar_ is a ratio between the integrated areas of the 1630 cm^−1^ and 2930 cm^−1^.

**Table 2:** The Inhibition rates of HAs and FAs against phytopathogenic fungi at 50 mg/kg.

Samples	Inhibition rate (%)								
	*P*.*P*	*B*.*C*	*R*.*C*	*F*.*G*	*P*.*I*	*S*.*S*	*R*.*S*	*C*.*H*	*B*.*M*
HA-CK	51.3 ± 0.6 a	38.3 ± 0.6 a	33.3 ± 1.5 a	33.7 ± 1.2 a	31.3 ± 1.5 a	26.7 ± 1.5 a	22.3 ± 1.2 a	24.3 ± 1.2 a	20.7 ± 1.2 a
HA-3a	16.3 ± 1.5 bc	38.3 ± 0.6 a	15.7 ± 1.2 bc	17.0 ± 1.0 b	13.7 ± 2.1 c	16.7 ± 0.6 b	11.3 ± 1.5 c	21.0 ± 1.0 bc	4.7 ± 1.5 bc
HA-6a	18.3 ± 1.2 b	24.7 ± 1.5 c	17.3 ± 0.6 b	13.7 ± 0.6 c	13.3 ± 0.6 c	11.0 ± 1.0 c	15.3 ± 0.6 b	18.7 ± 2.1 c	3.3 ± 0.6 c
HA-10a	6.3 ± 1.5 d	28.7 ± 1.2 b	14.3 ± 0.6 cd	17.7 ± 1.5 b	20.3 ± 1.5 b	11.3 ± 0.6 c	11.7 ± 2.1 c	22.0 ± 1.0 ab	6.3 ± 0.6 b
HA-20a	14.2 ± 2.0 c	14.3 ± 0.6 d	13.3 ± 0.6 d	13.3 ± 0.6 c	19.3 ± 0.6 b	11.3 ± 1.5 c	10.7 ± 0.6 c	13.7 ± 1.2 d	3.3 ± 0.6 c
FA-CK	29.7 ± 1.2 A	47.0 ± 1.0 A	23.3 ± 0.6 A	16.0 ± 1.0 A	21.7 ± 0.6 A	12.7 ± 1.2 A	19.7 ± 0.6 A	18.3 ± 0.6 A	11.3 ± 1.2 A
FA-3a	29.7 ± 0.6 A	46.7 ± 0.6 A	20.7 ± 0.6 B	13.3 ± 2.1 AB	15.3 ± 0.6 C	12.7 ± 0.6 A	20.0 ± 1.0 A	13.3 ± 0.6 B	6.3 ± 0.6 B
FA-6a	28.3 ± 0.6 A	22.3 ± 3.1 B	13.7 ± 1.2 D	13.0 ± 1.9 B	17.7 ± 1.5 B	11.3 ± 1.2 A	15.0 ± 1.0 C	8.7 ± 0.6 C	3.3 ± 0.6 C
FA-10a	15.7 ± 0.6 B	15.7 ± 0.6 C	16.3 ± 1.5 C	15.7 ± 0.6 AB	12.7 ± 0.6 D	11.7 ± 0.6 A	17.0 ± 1.0 B	8.3 ± 1.2 C	0 D
FA-20a	15.0 ± 1.0 B	24.3 ± 0.6 B	15.0 ± 1.0 CD	16.0 ± 1.0 A	14.3 ± 0.6 C	12.7 ± 0.6 A	10.7 ± 0.6 D	4.3 ± 0.6 D	0 D

Mean values and the standard deviation are given. Capital letters indicate significant differences between the humic acids, and small letters indicate significant differences between the fulvic acids. The significant differences are calculated by ANOVA with Ducan test (*P* < 0.05, n = 3), labeled from highest to lowest value.

**Table 3:** Relationships between the FT-IR peak areas and the inhibition rate against the tested phytopathogenic fungi revealed by Mantel test.

Peaks cm^−^	HAs inhibition rates		FAs inhibition rates	
	r	*P*	r	*P*
2930	0.309	0.038*	0.794	<0.001**
1720	0.121	0.177	0.792	<0.001**
1630	0.742	<0.001**	—	—
1540	−0.034	0.529	—	—
1450	0.707	<0.001**	0.067	0.157
1420	0.431	0.002**	0.421	<0.001**
1380	0.345	0.028*	0.028	0.311
1224	0.309	0.033*	0.719	<0.001**
1130	0.784	<0.001**	—	—
1040	−0.08	0.709	0.225	0.028*
Iar	0.593	<0.001**	—	—

The correlation (r) and significance (*P* value) were calculated. *P* value is from the Montel Carlo Permutation Tests. *Significant difference at 0.05 level, **Significant difference at 0.01 level.
