# Supplementary material for: Fungicidal activities of soil humic/fulvic acids as related to their chemical structures in greenhouse vegetable fields with cultivation chronosequence
Source: Sci Rep. 2016 Sep 6;6:32858. doi: 10.1038/srep32858 (PMC5011764; doi:10.1038/srep32858)
Supplement: Supplementary Information [file srep32858-s1.pdf]

Fungicidal activities of soil humic/fulvic acids as related to their chemical structures in greenhouse vegetable fields with cultivation chronosequence

Meng Wu[1], Mengya Song[1], Ming Liu[1], Chunyu Jiang[1], Zhongpei Li[1, 2, *]

[1]State Key Laboratory of Soil and Sustainable Agriculture, Institute of Soil Science, Chinese Academy of Sciences, Nanjing 210008, China

[2]Graduate University of Chinese Academy of Sciences, Beijing 100049, China

Soil samples	SOC (g kg^{-1})	TN (g kg^{-1})	TP (g kg^{-1})	TK (g kg^{-1})	AN (mg kg^{-1})	AP (mg kg^{-1})	AK (mg kg^{-1})	pH	C_{HA}/C_{FA}
CK	14.6±0.1 b	1.51±0.17 c	0.76±0.09 b	14.6±0.2 b	111.2±5.5 b	54.4±2.8 c	105.0±7.1 c	6.97±0.12 a	0.79±0.11 c
3a	20.7±3.2 a	2.45±0.20 a	1.32±0.40 ab	17.1±0.3 a	155.6±22.5 b	124.3±7.1 b	291.7±38.2 b	7.11±0.06 a	1.22±0.07 a
6a	21.8±1.3 a	2.38±0.01 a	1.01±0.02 ab	14.9±0.1 b	267.1±49.9 a	97.7±6.1 b	196.7±24.7 c	4.71±0.45c	1.03±0.06 b
10a	20.9±0.8 a	2.52±0.26 a	1.80±0.19 a	15.1±0.2 b	258.5±72.5 a	245.1±25.8 a	165.9±6.3 c	4.69±0.26 c	0.92±0.20 bc
20a	15.4±1.2 b	1.88±0.17 b	0.99±0.02 ab	17.2±0.6 a	155.6±27.8 b	95.6±14.4 b	336.7±13.7 a	5.47±0.08 b	0.89±0.04 bc

Supplementary Table S1. The chemical properties and C_{HA}/C_{FA} of the soils. Mean values ± SD are shown (n = 3), the significant differences are calculated by ANOVA with Duncan test ($P < 0.05$), labeled from highest to lowest value.
